# Regenerative potential of mesenchymal stromal cells in wound healing: unveiling the influence of normoxic and hypoxic environments

**DOI:** 10.3389/fcell.2023.1245872

**Published:** 2023-10-10

**Authors:** Mohamad Mahjoor, Arshia Fakouri, Simin Farokhi, Hojjatollah Nazari, Hamed Afkhami, Fatemeh Heidari

**Affiliations:** ^1^ Cellular and Molecular Research Center, Qom University of Medical Sciences, Qom, Iran; ^2^ Department of Immunology, Faculty of Medicine, Iran University of Medical Sciences, Tehran, Iran; ^3^ Student Research Committee, USERN Office, Lorestan University of Medical Sciences, Khorramabad, Iran; ^4^ School of Biomedical Engineering, University of Technology Sydney, Sydney, NSW, Australia; ^5^ Nervous System Stem Cells Research Center, Semnan University of Medical Sciences, Semnan, Iran; ^6^ Department of Medical Microbiology, Faculty of Medicine, Shahed University, Tehran, Iran; ^7^ Department of Anatomy, Faculty of Medicine, Qom University of Medical Sciences, Qom, Iran

**Keywords:** hypoxic MSCs, mesenchymal stromal/stem cells (MSCs), normoxic MSCs, wound, wound healing

## Abstract

The innate and adaptive immune systems rely on the skin for various purposes, serving as the primary defense against harmful environmental elements. However, skin lesions may lead to undesirable consequences such as scarring, accelerated skin aging, functional impairment, and psychological effects over time. The rising popularity of mesenchymal stromal cells (MSCs) for skin wound treatment is due to their potential as a promising therapeutic option. MSCs offer advantages in terms of differentiation capacity, accessibility, low immunogenicity, and their central role in natural wound-healing processes. To accelerate the healing process, MSCs promote cell migration, angiogenesis, epithelialization, and granulation tissue development. Oxygen plays a critical role in the formation and expansion of mammalian cells. The term “normoxia” refers to the usual oxygen levels, defined at 20.21 percent oxygen (160 mm of mercury), while “hypoxia” denotes oxygen levels of 2.91 percent or less. Notably, the ambient O_2_ content (20%) in the lab significantly differs from the 2%–9% O_2_ concentration in their natural habitat. Oxygen regulation of hypoxia-inducible factor-1 (HIF-1) mediated expression of multiple genes plays a crucial role in sustaining stem cell destiny concerning proliferation and differentiation. This study aims to elucidate the impact of normoxia and hypoxia on MSC biology and draw comparisons between the two. The findings suggest that expanding MSC-based regenerative treatments in a hypoxic environment can enhance their growth kinetics, genetic stability, and expression of chemokine receptors, ultimately increasing their effectiveness.

## Graphical Abstract

**Figure:**
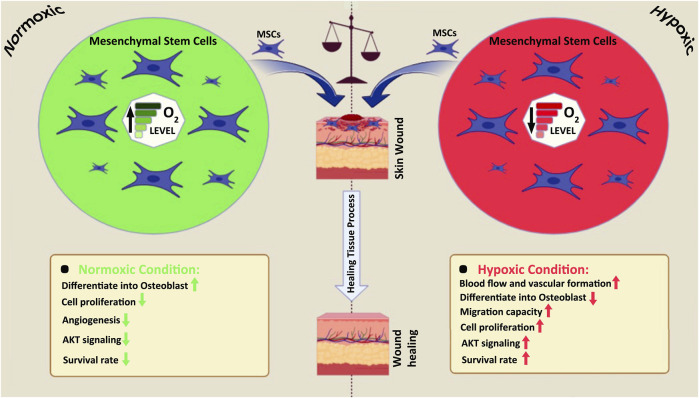
The differences between normoxic and hypoxic MSCs on wound healing.

## 1 Introduction

The skin’s ability to heal wounds results from the body’s innate ability to repair and regenerate damaged tissues (Lau et al., 2017). It is a common occurrence in people due to accidents, surgeries, burns, and long-term illnesses. Various wound therapies have been suggested, such as pharmacotherapy (Jackson et al., 2012; Abdullahi and Jeschke, 2014), cellular therapy (Nauta et al., 2013; Morimoto et al., 2015), extracorporeal shock wave therapy (Mittermayr et al., 2012; Webster et al., 2012), negative pressure wound therapy (NPWT) (Webster et al., 2012), electrical stimulation therapy (Thakral et al., 2013; Baleg et al., 2015), and light therapy (Haubner et al., 2012; Lau et al., 2015).

The use of mesenchymal stromal/stem cells (MSCs) to treat skin wounds has gained popularity. MSCs offer a reasonable, safe, and successful therapeutic method due to their differential competency and other features, including ease of collection, low immunogenicity, and their essential role in native wound healing physiology (Zou et al., 2012; Markov et al., 2021; Hassan et al., 2014). In fact, MSCs promote the migration of skin cells, the development of blood vessels, the re-epithelialization of wounds, and the production of granulation tissue, thereby accelerating the healing process. Remarkably, they foster a regenerative wound-healing environment instead of a fibrotic one (Zahorec et al., 2015; Yao et al., 2016). While clinical studies have provided evidence suggesting the safety, practicality, and efficacy of MSC-based therapies (Huang et al., 2020; Gentile and Garcovich, 2021; Mahjoor et al., 2023), more evidence is needed due to small sample sizes and a lack of long-term follow-up. Multipotent stem cells (MSCs) exert therapeutic effects through various cell type-specific and wound-healing phase-specific functions, from hemostasis to remodeling (Hocking and Gibran, 2010).

Many adult organs, including bone marrow, adipose tissue, and umbilical cords (UCs), contain mesenchymal stem cells (MSCs) (Pereira et al., 2013). MSCs typically undergo two distinct environments along their journey from isolation to engraftment. Both the *in vivo* or physiological setting and the *in vitro* culture setting (from isolation to transplanting) are considered (before isolation and after transplantation). Cells are exposed to 20% O_2_ during most MSC growth techniques nowadays, which is around 4–10 times higher than the concentration of O_2_ in their natural habitats (Chow et al., 2001; Antoniou et al., 2004). MSCs cultivated *in vitro* may be stressed by the increased oxygen levels. Moreover, various studies have provided significant evidence in recent years, indicating the deleterious effect of ambient O_2_ concentration on MSCs, including early senescence, prolonged population doubling time, DNA damage (Fehrer et al., 2007; Estrada et al., 2012), and poor engraftment upon transplantation (Sch¨achinger et al., 2006a). These findings highlight the critical influence of O_2_ concentration on the biology of MSCs. Over the past 2 decades, *in vitro* research examining the pathways involved in stem cell preservation has proliferated. However, the effects of normoxic (often 20%–21% O_2_ concentration) and hypoxic (typically 2%–9% O_2_ concentration) environments on stem cell development have been largely overlooked (Simon and Keith, 2008). This article compares and contrasts MSC culture under normoxic and hypoxic *in vitro* settings. Lastly, this research discusses how MSCs cultivated *in vitro* under hypoxic settings might serve as a therapeutic option.

## 2 Wound and wound healing

Wounds on the skin can be classified into two categories: those requiring tissue regeneration and those that do not. Open wound edges are brought together, and the wound is mostly closed through localized matrix remodeling after surgery or a minor incision. However, a complicated chain reaction can occur when tissue loss is significant in wounds, such as burns, abrasions, and traumatic wounds.

The first phase involves hemostasis and inflammation, which helps rebuild a sterile barrier and reduces exposure to the outside world. Subsequently, the wound undergoes tissue replacement (scar formation). If difficulties arise during the early phases of healing, such as an inadequate immune response or impaired angiogenesis, wounds can become chronic or non-healing (Lazarus et al., 1994; Yates et al., 2011; Frykberg and Banks, 2015; Mahmoudvand et al., 2023). Chronic wounds encompass vascular ulcers, pressure ulcers, and diabetic ulcers, and each patient requires a customized treatment plan based on the severity of their condition. Lack of wound healing progress poses risks of infection, tissue spread, and potential amputation.

To combat the severe consequences of chronic wounds, scientists and doctors are diligently developing and implementing innovative biological treatments. Abnormalities in the last two phases of wound healing, such as keloid or hypertrophic scarring, may result in extensive scarring in contrast to chronic wounds. Although the epithelium may regenerate, and the wound may appear healed, the underlying dermis in these scars is damaged and dysfunctional. Improperly healed wounds tend to be structurally weaker and more susceptible to re-ulceration.

Healing a wound involves a complex synchronization of several cell types, signaling pathways, and microenvironmental adjustments across three overlapping stages of repair: hemostasis/inflammation, tissue replacement, and resolution. The initial step is the hemostasis/inflammation phase, during which the focus is on stopping bleeding from broken blood vessels and preventing the spread of pathogenic infection. Platelets in the bloodstream join with enzymatically transformed fibrin to create a clot that covers the wound and acts as a temporary matrix (Barker and Engler, 2017). This fibrin clot is physiologically active and recruits pro-inflammatory macrophages (M1) and leukocytes, triggering a localized immune response in addition to preventing infection and clearing the wound of cellular and extracellular debris. The tissue replacement phase begins when specialized cells, such as fibroblasts, endothelial cells, epidermal cells, and progenitor cells, enter the wound bed. Fibroblasts produce collagen III-rich granulation tissue to temporarily replace the missing extracellular matrix (ECM), endothelial cells generate new blood vessels through angiogenesis, and epidermal cells migrate beneath the scab to permanently seal the wound surface and restore the epidermis. At this stage, pro-inflammatory macrophages (M1) decrease and are replaced by wound-healing macrophages, categorizing the lesion as sterile (M2). These M2 macrophages facilitate additional vascular healing by producing and modulating the granulated ECM.

The resolution phase involves the permanent replacement of the wound bed with mature ECM, removal of up to 90% of the surplus blood vessels, and cessation of any remaining proliferation and migration signals, replaced with stop signals, such as those operating via CXCR3 (Bodnar et al., 2013; Martins-Green et al., 2013; Mirshekar et al., 2023). Transdifferentiation of fibroblasts into myofibroblasts causes the wound bed to contract by reorganizing and restructuring juvenile collagen III into mature collagen I, thereby restoring the skin’s tensile strength (Sahota et al., 2003; Mahjoor et al., 2021). If everything proceeds as expected, the final result will be little more than a barely noticeable scar.

## 3 Mesenchymal stem cells (MSCs)

Various types of stem cells, including bone marrow mesenchymal stem cells (BM-MSCs), umbilical cord MSCs, adipose-derived stem cells (ASCs), molar MSCs, and amniotic fluid ASCs, have been investigated as potential sources of MSCs, which hold significant promise in the field of stem cell therapies. These MSCs, regardless of their source, facilitate the healing process through two main processes: paracrine effects and direct development into skin cells (fibroblasts and keratinocytes) (Martins-Green et al., 2013; Kinnaird et al., 2004).

Miao *et al.* conducted a comparison between placental-derived MSCs and bone marrow-derived MSCs, assessing their morphology, growth, membrane markers, and differentiation potential (Yao et al., 2016; Lau et al., 2017). Placental MSCs exhibited similar morphology and growth characteristics, as well as the presence of markers CD105, CD29, and CD44. Notably, they lacked expression of hematopoietic markers CD34, CD45, and HLA-DR. Furthermore, the study demonstrated that placental MSCs could differentiate into endothelial and neuronal cells (Tsai et al., 2012). In another recent study focusing on venous leg ulcer treatment, allogeneic AM transplantation proved highly beneficial for serious wounds, showing antiadhesive properties, wound protection, and effective re-epithelialization (anti-scarring) (Martin-Rendon et al., 2007a).

MSCs secrete a myriad of active compounds, such as insulin-like growth factor, hepatocyte growth factor, transforming growth factor beta-1, vascular endothelial growth factor (VEGF), keratinocyte growth factor, fibroblast growth factor 2, platelet-derived growth factor, and collagen I9, which play critical roles in wound healing by stimulating angiogenesis, skin cell proliferation, and ECM formation (Fui et al., 2019). The precise mechanism of direct differentiation of MSCs into specific skin cells remains incompletely understood. However, it is well-established that MSCs have the capacity to differentiate into various types of resident cells when introduced into a wound site. The primary mechanism through which stem cells differentiate into specialized cell types, such as endothelial cells, fibroblasts, and keratinocytes, is through the local release of multiple chemicals. Thus, strategies aimed at augmenting the paracrine MSC secretion within the wound microenvironment hold the potential to enhance the efficiency of wound healing. One promising approach could involve modifying the composition of the suspension by utilizing bubbles encapsulating a thoughtfully selected secretome, which can then be applied to the wound to potentially enhance wound healing outcomes. However, randomized trials confirming the effectiveness and safety of MSC use in wound treatment are still needed. Clinical trials involving MSCs are currently underway, and their findings are awaited to further validate the effectiveness and safety of MSC-based treatments for wound healing (Kyung-Chul et al., 2019).

In the process of wound healing, MSCs originating from subcutaneous fat tissue and the blood supply play a role in regulating early and mid-phase inflammation, as well as contributing to the restoration of dermal tissue. Upon infiltrating the wound, MSCs initiate the secretion of proinflammatory cytokines, which recruit neutrophils and M1 converted macrophages, aiding in the degradation of damaged tissue during the hemostasis/inflammation phase. Additionally, MSCs help in controlling the overall inflammatory response by reducing the number of activated T cells, neutrophils, and macrophages. During the tissue replacement phase, MSCs guide the polarization of monocytes into proreparative M2 macrophages, facilitating the clearance of cellular debris and modifying the temporary matrix. In the resolution phase, MSCs continue to regulate the matrix by releasing matrix metalloproteinases (MMPs) and tissue inhibitors of metalloproteinases (TIMPs), while also maintaining a balance between transforming growth factor-b1 and transforming growth factor-b3 to prevent hypertrophic scarring (Yates et al., 2011).

A unique delivery system employing fibrin glue was investigated in both acute and chronic wounds by Falanga *et al.* (Sch¨achinger et al., 2006b). They topically applied bone marrow-derived MSCs combined with a fibrin spray up to three times. The study observed that surgical defects from excision of nonmelanoma skin cancers healed within 8 weeks, suggesting that MSCs contributed to accelerated resurfacing. Additionally, chronic lower-extremity wounds present for longer than 1 year significantly decreased in size or healed completely by 20 weeks. The study also found a correlation between the surface density of MSCs and the reduction in ulcer size. Another study by Yoshikawa *et al.* included 20 patients with various nonhealing wounds who were treated with autologous bone marrow-derived MSCs cultured on a collagen sponge (Chavakis et al., 2010). It was observed that 90% of the wounds healed completely when treated with the cell composite graft, and the addition of MSCs facilitated tissue regeneration.

In 1958, Cooper *et al.* and Zwartouw & West-wood noticed that certain cells grew more quickly at low O_2_ tension levels compared to standard air levels (Cooper et al., 1958; Zwartouw and Westwood, 1958). This prompted them to conduct the first experiments to evaluate the effects of varied O_2_ tension levels on MSC growth. Hypoxia has been identified as a key regulator of MSC recruitment, migration, and differentiation (Raheja et al., 2010a; Raheja et al., 2011). A study by Rochefort *et al.* demonstrated that hypoxia mobilized MSCs from the bone marrow into the peripheral blood but not hematopoietic progenitor cells (Rochefort et al., 2006). Additionally, stem-cell quiescence and plasticity have been linked to low O_2_ tension levels (D’Ippolito et al., 2006; Liu et al., 2011). Researchers have been interested in finding the appropriate O_2_ tension levels for *in vitro* cultivation (Das et al., 2010). Raheja *et al.* (2010) conducted an experiment in which they seeded and stimulated MSCs for differentiation in different oxygen concentrations. Results indicated that 21% oxygen was optimal for MSC development into osteoblasts, whereas oxygen levels below 5% significantly slowed the differentiation process. At 5% and 21% O_2_, however, no significant change in osteogenic markers was detected (Raheja et al., 2010b). Early passaged (P2) MSCs at 5% O_2_ concentration showed enhanced osteoblastic and adipogenic differentiation capacity (Basciano et al., 2011a). Recent studies have shown that MSCs may survive in an oxygen-depleted (1%–5% O_2_) environment (Grayson et al., 2007a; Nekanti et al., 2010; Holzwarth et al., 2010a; L´opez et al., 2011). It has also been observed that MSCs isolated from adipose tissue that has been precultured in a hypoxic environment have enhanced adipogenic and osteogenic differentiation potentials (Valorani et al., 2012) (Table 1). Nevertheless, when MSCs were maintained and driven for differentiation in 1% O_2_ concentration, only a small number of studies demonstrated a loss in differentiation potential (Yang et al., 2011; Hung S.-P. et al., 2012). Several clinical trial data indicate that transplantation of MSCs cultivated under an ambient environment has a limited ability for engraftment (Mohamadnejad et al., 2010; Sch¨achinger et al., 2006b).

**TABLE 1:** The differences between normoxic and hypoxic MSCs.

Cell type	Differences	Ref
*Normoxic MSCs*	- Differentiate into osteoblast most rapidly	Raheja et al. (2010b)
	- Survival rate is Low compared to hypoxic conditions	Wang et al. (2008a), Hu et al. (2008)
	- Degrade phosphorylation of the Akt signaling pathway	Hill et al. (2009)
	- Cell proliferation rate is Low compared to hypoxic conditions	Grayson et al. (2007b)
	- Transcriptional activity of hypoxia inducible factor-1 (HIF-1) is ubiquitinated and degraded and reduce the angiogenesis	Hill et al. (2009)
*Hypoxic MSCs*	- Reduce differentiate into osteoblast	Raheja et al. (2010b)
	- Increased adipogenic and osteogenic differentiation	Basciano et al. (2011a), Valorani et al. (2012)
	- Enhanced skeletal muscle regeneration, improved blood flow and vascular formation	Leroux et al. (2010)
	- Expression of chemokine receptors CXCR4, CXCR7, and CX3CR1 was upregulated (play an important role in damaged-tissue-specific trafficking and homing of MSCs)	Hung et al. (2007a), Liu et al. (2012a), Saller et al. (2012), Tsai et al. (2012)
	- Increased level of glucose resulting from gluconeogenesis	Ren et al. (2006a), Martin-Rendon et al. (2007a), Hung et al. (2007b)
	- Overexpression of HIF-1, increase in erythropoietin receptors, and anti-apoptotic factors Bcl-2 and Bcl-XL, followed by decreased Caspase-3 levels	Wang et al. (2008a), Hu et al. (2008)
	- Increase phosphorylation of the Akt signaling pathway	Hill et al. (2009)
	- Increased cell proliferation rate	Grayson et al. (2007b), Martin-Rendon et al. (2007b)
	- Enhanced the migration capacity	Hung et al. (2012c)
	- Decreased the time necessary for the cell population to double and increase in the number of cells in the G2/S/M phase	D’Ippolito et al. (2006), Ren et al. (2006b)
	- Increased secretion of VEGF and (MT1-MMP), reduced levels of (MMP2)	Wu et al. (2007)
	- Decreased expression of TGF-β3, increased expression of FGF2 and VEGF	Potier et al. (2007)

Clinical studies utilizing MSCs have shown some promising therapeutic results, as documented in various review papers and meta-analyses (Abdel-Latif et al., 2007; Lipinski et al., 2007; Chavakis et al., 2010; Song et al., 2011). The engraftment and differentiation capacity to cardiomyocytes *in vivo* of first-passage mouse BM-MSCs was shown to be higher than that of fifth-passage mouse BM-MSCs (Jin et al., 2011). Moreover, murine MSCs pre-conditioned in a hypoxic environment demonstrated increased blood flow and vascular development at day 7 compared to MSCs maintained in a normoxic condition (Leroux et al., 2010). Additionally, hypoxia or a reagent that mimics the reaction to hypoxia increased the expression of the chemokine receptors CXCR4, CXCR7, and CX3CR1 (Hung S.-C. et al., 2007; Liu et al., 2012a; Saller et al., 2012; Tsai et al., 2012). Survival of MSCs in hypoxic environments depends on their capacity, shared by all cells, to switch efficiently from aerobic to anaerobic metabolic pathways (Das et al., 2010). An experimental investigation done with rats on the alterations in MSCs under serum-deprivation and hypoxia settings indicated that serum deprivation was the critical factor leading to ischemia-induced death of MSCs. Nevertheless, it also indicated that prolonged exposure to hypoxia led to mitochondrial malfunction and Caspase-3 activation, a crucial factor in apoptosis (Zhu et al., 2006). The buildup of lactate from glycolysis may become an inhibiting factor over the long term (Lord-Dufour et al., 2009; Das et al., 2010), despite the fact that MSCs have been proven to resist hypoxia (e.g., O_2_ 1%) for at least 48 h (Martin-Rendon et al., 2007a). Hypoxia-inducible factor-1 alpha (HIF-1α), when stabilized, migrates into the cell nucleus and joins with hypoxia-inducible factor-1 beta (HIF-1β) to promote intracellular signaling pathways linked with cell survival. To regulate gluconeogenesis, hypoxia-responsive genes such as glucose-6-phosphate transporter (G6PT) bind to their promoter regions as dimeric complexes. There is evidence that the higher glucose level from gluconeogenesis aids MSC survival in hypoxic or serum-depleted environments (Ren et al., 2006a; Martin-Rendon et al., 2007a; Hung S. C. et al., 2007). In addition to HIF-1 upregulation, a rise in erythropoietin receptors and anti-apoptotic proteins Bcl-2 and Bcl-XL, followed by lower Caspase-3 levels, all contribute to a higher survival rate under hypoxia compared to normoxia. In addition, hypoxia stimulates the production of the proangiogenic molecules interleukin IL-6 and VEGF (Wang Y. et al., 2008) (Table 1). Several signaling pathways, such as the Akt and ERK pathways, control these positive effects.

The Akt signaling pathway is phosphorylated in response to hypoxia and HIF-1 stabilization but is destroyed in the presence of normal oxygen levels. The expression of the pro-apoptotic protein Bax is downregulated, while the expression of the anti-apoptotic factor Bcl-2 is upregulated when Akt is active. Apoptosis or additional stabilization of HIF-1, leading to its translocation into the cell nucleus and activation of hypoxia-responsive genes like G6PT and angiogenesis-related proteins like VEGF and IL-6, may result from such overexpression interacting with the Bax accumulating in the mitochondria (Hill et al., 2009). The ideal culture time of MSCs in hypoxic circumstances, like the optimal O_2_ tension level, is yet unknown. After just 10 min in culture, Wang *et al.* found that MSCs subjected to short-term hypoxia pre-conditioning showed improved cell survival and angiogenic characteristics (Wang J. A. et al., 2008). Cell growth was shown to be 30 times greater in low O_2_ conditions (2% for 7 passes) compared to normoxic cultures by Grayson *et al.* (Grayson et al., 2007b). After 7 days in culture, Hung *et al.* found that MSCs could proliferate more effectively when exposed to hypoxia (1% O_2_) (Hung et al., 2012b). An *in vitro* migration experiment was also carried out in this work, and the results revealed that MSCs’ migratory potential was improved by hypoxia (Hung et al., 2012c). Matin-Rendon *et al.* found that MSC proliferation increased even after just 24 h of exposure to 1.5% O_2_ (Martin-Rendon et al., 2007b). The number of cells in the G2/S/M phase was shown to rise during hypoxia (Ren et al., 2006b), and D'Ippolito *et al.* demonstrated that a low O_2_ tension level shortened the time required for the cell population to double when cultivated at 3% O_2_ (D’Ippolito et al., 2006).

The opposite was found in research by Holzwarth *et al.*, which compared the proliferation rates of MSCs after 7 days of culture at 21%, 5%, 3%, and 1% O_2_. Strong proliferation rates were seen in their research when MSCs were cultured at 21% O_2_, which is hyperoxic compared to the physiological environment in which MSCs dwell. After 7 days in culture, a significantly lower percentage of MSCs (1.37%) reached the G2/M phase in hypoxic cell cultures (1% O_2_) than in cultures with 21% O_2_. With fewer cells in the G2/M phase, the authors conclude that cell proliferation is inhibited under low- O_2_ conditions (Holzwarth et al., 2010b). Via nuclear factor kappa-dependent processes (Crisostomo P. R. et al., 2008), hypoxia seems to control the concentrations of soluble factors (including VEGF, fibroblast growth factor-2 (FGF2), hepatocyte growth factor, and insulin-like growth factor 1 (IGF-1)). Results indicate that hypoxia may have an effect on the immunoregulatory capacities of MSCs. After 24 h of exposure to hypoxia, Wu *et al.* found that most genes were regulated using human MSCs. Nevertheless, matrix metalloproteinase-2 (MMP2) levels dropped after less than 4 h of hypoxia, but VEGF and membrane type 1-matrix metalloproteinase (MT1-MMP) levels increased (Wu et al., 2007). Increased VEGF expression was also reported by Muir *et al.* in hypoxic circumstances (Muir et al., 2006). After 48 h of cultivation with FBS under hypoxia, Potier *et al.* found that the expression of TGF-3, but not FGF2 or VEGF, was reduced. The levels of IL-6, IL-8, and MPC1 were not altered in that investigation (Potier et al., 2007). In contrast, Hung *et al.* reported that under hypoxia, the expression of IL-6, macrophage chemotactic protein (MCP1), and VEGF was elevated in MSCs grown in an FBS-free media (Hung S. C. et al., 2007).

Nakagawa *et al.* suggested that MSCs, together with bFGF in a skin defect model, accelerate wound healing and showed that the human MSCs transdifferentiated into the epithelium in rats (Valorani et al., 2012). Shumakov *et al.* showed that the transplantation of MSCs on the surface of deep burn wounds in rats decreased inflammatory cell infiltration and accelerated the formation of new vessels and granulation tissue (Hung S.-P. et al., 2012). The cells were also shown to produce bioactive substances that seemed to accelerate the regeneration process. Collectively, these data demonstrate that MSC treatment impacts all phases of wound repair, including inflammation, epithelialization, granulation tissue formation, and tissue remodeling.

The chemokine receptors CX3CR1 and CXCR4 and the hepatocyte growth factor receptor cMet are all regulated by hypoxia, which also affects the production of soluble factors. These receptors enhance MSCs’ ability to migrate to and home in on injured cells (Das et al., 2010). Hepatocyte growth factor and its receptor cMet, the expression of which is elevated during hypoxia, may have a role, as discovered by Rosova *et al.* (Rosova et al., 2008a). Hung *et al.* demonstrated that MSCs’ migratory ability was enhanced by hypoxia, and Wang *et al.* demonstrated the same thing in the context of a brain injury (Hung et al., 2012d). The CXCR4 receptor was implicated in the capacity of these cells to move to injured tissue in the latter investigation (Wang Y. et al., 2008). In animal models, MSCs cultured under hypoxia appear to have improved immune regulatory performance, suggesting a possible role of hypoxia in cellular therapy strategies (Mao et al., 2004; Hu et al., 2008). Hypoxia has also been shown to influence the secretion of trophic factors and membrane markers associated with MSC migration and homing. Several cytokines, chemokines, and integrins may all play a role in migration. One chemokine that is widely expressed is stromal cell-derived factor-1 (SDF-1/CXCR4 receptor) (Liu et al., 2012b). Thus, the expression of CXCR4 on the surface of MSCs cultivated under hypoxia has a significant role in the migration of transplanted cells (Martin-Rendon et al., 2007b).

Others defined a hypoxia pretreatment of tissue-engineered substitutes, which are 3D biomimetic collagen-chitosan sponge scaffolds (CCSS), as biocompatible and biodegradable supports for the effective delivery, favorable adhesion, and survival of BM-MSCs to accelerate chronic wound healing by inhibiting inflammation and improving angiogenesis (Lazarus et al., 1994; Yates et al., 2012). 3D biomimetic tissue-engineered substrates have a great effect on the development of scaffolds for tissue engineering in chronic wound healing, particularly in combination with cells such as MSCs, fibroblasts, keratinocytes, and endothelial cells. Co-transplantation of CD341 cells with CD341-derived endothelial cells in a 3D fibrin scaffold improved wound healing by decreasing the inflammatory reaction and increasing neovascularization in diabetic rats. The relative secreted factors include VEGF, PDGF, bFGF, IL-10, and IL-17 (Lau et al., 2015). In addition, a similar tissue-engineered substitute using autologous fibroblasts and keratinocytes seeded in Hyaff-11, an ester of hyaluronic acid, has been reported to enhance wound healing in diabetics and is associated with enhanced revascularization (Barker and Engler, 2017).

Others found that the major inflammatory cell infiltrated the CCSS in the wound site, and the expression of TNF-a and IL-6 was significantly higher at early implantation. However, BMMSCs efficiently improved this effect and significantly suppressed TNF-a and IL-6 levels in the wound site, thereby contributing to improved wound healing, particularly, hypoxia pretreatment of the tissue-engineered substitute. They found that the expression of IL-10 was enhanced at the wound site and that IL-10 levels in the tissue-engineered substitute with hypoxia pretreatment were higher compared with other conditions. In addition, normoxic pretreatment of the tissue-engineered substitute also caused a significant increase in IL-10 levels when compared with CCSS only in the wound site.

### 3.1 Normoxic and hypoxic MSCs in wound repair

The molecular signaling molecule oxygen profoundly influences the formation and behavior of mammalian cells. Oxygen deprivation (hypoxia) exerts diverse effects on different cell types, leading to alterations in proliferation (Packer and Fuehr, 1977), adhesion (Lash et al., 2001), apoptosis (Carmeliet et al., 1998), metabolism (Loike et al., 1992), ECM secretion (Horino et al., 2002), growth factor expression (Minchenko et al., 1994), and differentiation phenotypes (Lennon et al., 2001) (Graphical Abstract). The paracrine activity of MSCs induces the overexpression of several secretable factors, including VEGF, transforming growth factor-beta 1 (TGF-1), among others, which may result in apoptosis under hypoxic conditions (Crisostomo P. R. et al., 2008; Lee E. Y. et al., 2009). In comparison to AD-MSCs cultured in normoxic conditioned medium (norCM), AD-MSCs cultured in a hypoxic conditioned medium (hypoCM) have been shown to enhance human dermal fibroblast migration and decrease wound area in an *in vivo* model (Table 2) (Lee E. Y. et al., 2009). Many MSCs reside in a low oxygen tension environment (between 3% and 9%), depending on the tissue source, and hypoxia within the stem cell niche appears to sustain stem cells’ ability to self-renew, proliferate, and migrate, all of which are essential for their therapeutic efficacy (Rosova et al., 2008b; Ivanovic, 2009; Basciano et al., 2011b; Kimura and Sadek, 2012; Ejtehadifar et al., 2015; Zhou et al., 2022). However, when oxygen levels drop to deficient levels (1.5% O_2_), as is often the case in a wound bed, MSCs may become overstressed and undergo apoptosis (Ejtehadifar et al., 2015).

**TABLE 2:** Effects of normoxic and hypoxic MSCs on wound healing.

MSCs	Wound healing	Ref
Normoxic	- Promoted primary keratinocyte wound healing	Walter et al. (2010), Lee et al. (2012)
	- Transcriptional activity of hypoxia inducible factor-1 (HIF-1) is ubiquitinated and degraded and reduce the angiogenesis	Semenza (2003)
	- Release different soluble factors such KGF,EGF, IGF-1,VEGF-A, PDGF, EPO and TPO that increase fibroblast, keratinocyte and endothelial cell migration and increase extracellular matrix, which eventually accelerate wound healing	Chen et al. (2008), Chen et al. (2014b), Lee et al. (2016)
Hypoxic	- Promoted the migration of human dermal fibroblasts, and obviously reduced the wound area	Lee et al. (2009a)
	- Enhanced the migration and proliferation of keratinocytes and fibroblasts *in vitro*, and the migration of CD14^+^ monocytes	Xie et al. (2008)
	- Increase endothelial cells proliferation and migration, promoting early events of angiogenesis	Xie et al. (2008)
	- Accelerated wound closure	Xie et al. (2008)
	- Elevated levels of HIF-inducible secreted molecules other than bFGF and VEGF-A	Xie et al. (2008)
	- Enhance the proliferation, recruitment of skin cells after injury, neovascularization, angiogenesis, cell proliferation and recruitment of resident macrophages, regulation of collagen synthesis	Xie et al. (2008)
	- HIF-1α gets accumulated and forms a heterodimer with HIF-1β which results in transcription of genes involved in angiogenesis, cell proliferation, cell survival, cell migration and apoptosis	Semenza (2003)
	- Release different soluble factors such KGF, EGF, IGF-1, VEGF-A, PDGF, EPO and TPO that increase fibroblast, keratinocyte and endothelial cell migration and increase extracellular matrix, which eventually accelerate wound healing	Chen et al. (2008), Chen et al. (2014b), Lee et al. (2016)
	- Improved wound repair by secreting the IL-6 and IL-8	Chen et al. (2014c)

Hypoxic BM-MSCs secrete conditioned medium (hypoCM) that promotes the proliferation and migration of keratinocytes and fibroblasts *in vitro*, as well as the migration of CD14^+^ monocytes (Table 2). In contrast, norCM hardly influences the growth of these cells in any significant manner. HypoCM can also boost endothelial cell proliferation and migration, thus stimulating the initial stages of angiogenesis (Table 2). Angiogenesis, cell proliferation at the injury site, and recruitment of local macrophages were significantly increased by hypoCM compared to vehicle control medium and norCM, leading to expedited wound closure *in vivo*. HypoCM likely contains increased amounts of secreted molecules, including bFGF and VEGF-A, induced by HIF, which contributes to the acceleration of wound healing by increasing angiogenesis, cell proliferation, and the recruitment of resident macrophages to the wound. HypoCM also regulates collagen synthesis/degradation and alters collagen composition at the injury site, potentially acting synergistically with bFGF and VEGFA (Table 2). The quality of wound healing was shown to be higher in the hypoCM group. This occurrence may be due to the alternative and regulatory actions of bFGF (Xie et al., 2008), which are brought about by hypoxia when considering the unchanging gene expression level of MMP-1 and the undifferentiated or low protein expression and/or secretion of TGF-b1-3. Based on these findings, regenerative medicine procedures using hypoxic BM-MSCs and their released products may be employed to improve tissue healing after subcutaneous damage (Chen et al., 2014a). It was evident that ASCs cultured under hypoxic circumstances released substances that promoted wound healing *in vitro* using primary keratinocytes. This impact was amplified compared to normoxic settings. Wound repair mediated by keratinocytes is greatly aided by ASCs and other MSCs (Table 2) (Walter et al., 2010; Lee et al., 2012). Primary human keratinocytes, which are more representative of the human body than immortalized cell lines, show the same impact. It is interesting to note that the irregular cellular migration seen during wound closure may be an indicator of epithelial-to-mesenchymal transition in keratinocytes (Moreno-Bueno et al., 2009).

Hypoxia can alter the production of inflammatory and vasculogenic cytokines in mono- and co-culture, and the conditioned media acquired under hypoxia can stimulate wound healing and vascular development *in vitro*. Hypoxia-inducible factor-1 (HIF-1) is ubiquitinated and destroyed when oxygen levels are normal, preventing it from enacting its transcriptional activity. To counteract this, HIF-1 accumulates under hypoxic circumstances, forming a heterodimer with HIF-1 and triggering the transcription of genes involved in angiogenic processes, cell proliferation, survival, migration, and death (Table 2) (Semenza, 2003). The conditioned media (CM) obtained from MSC cultivation *in vitro* includes many cytokines, chemokines, and growth factors (Burdon et al., 2011), and several studies have shown that the paracrine actions of MSC have regenerative potential (Burdon et al., 2011). Angiogenic factors, including VEGF-A, MCP-1, and angiogenin, have been found in the CM from MSC cultivated in hypoxia (Hung SC. et al., 2007). Researchers have also looked into the paracrine effect of MSC. Their findings suggest that MSC grown in normoxia and hypoxia release different soluble factors like KGF, EGF, IGF-1, VEGF-A, PDGF, EPO, and TPO that increase fibroblast, keratinocyte, and endothelial cell migration and increase extracellular matrix, which speeds up wound healing (Table 2). The normoxic co-culture group had higher levels of the pro-inflammatory cytokines IL-1, IL-6, and IL-8 than the NMSC group. However, HMSCs had lower levels of IL-1 and IL-8 than NMSCs did. Pre-conditioning MSC with hypoxia may decrease their immunoreactivity by lowering the expression of pro-inflammatory genes.

Conversely, in both the normoxia and hypoxia groups, the addition of EC boosted the short-term expression of pro-inflammatory cytokines, indicating more significant immunoreactivity from co-culturing the two cell types. Hypoxic groups showed increased VEGF-A gene and protein expression. Several investigations have shown the same (Chen et al., 2008), and it is well established that MSC expresses considerably more VEGF-A than EC *in vitro* (Pedersen et al., 2014). While the paracrine impact of MSC has been demonstrated to trigger the sprouting of EC through VEGF signaling (Beckermann et al., 2008; Sordi et al., 2005), our results imply that VEGF-A production is enhanced by hypoxia regardless of the presence of EC.

In *in vitro* and animal experiments, hypoxia was shown to boost ADSC proliferation and improve ADSC wound-healing capability. Hypoxia-induced upregulation of growth factors VEGF and bFGF in ADSCs may be involved in the process (Lee EY. et al., 2009; Li et al., 2023). Because of their ability to stimulate angiogenesis, cell migration, proliferation, and other processes necessary for wound healing, hypoxic BM-MSCs release various growth factors and cytokines. Moreover, IL-6 and IL-8, produced by BM-MSCs, should also be involved in the enhanced wound healing caused by hypoCM (Table 2) (Chen et al., 2014c), due to their promotive effects on the function of fibroblasts and skin cell migration. The angiogenic growth factor ANG-1 has been linked to healthy blood vessel function and integrity (Suri et al., 1996). Several researchers found that hypoxia suppressed ANG-1 expression while elevating ANG-2 expression in co-cultures maintained under normal and lowered oxygen levels. Hypoxia increases ANG-2 expression in endothelial cells (Simon et al., 2008), while reducing ANG-1 expression (Enholm et al., 1997). The ANG-1/ANG-2 ratio is downregulated by hypoxia, which may result in immature vessel development during angiogenesis (Lund et al., 2004). The oxygen tension of the tissue around a wound plays a mediating role in the healing process. As a wound forms, hypoxia occurs as a normal part of the healing process. Resident MSCs move to the injury site after being drawn there by the favorable microenvironment, aiding in wound healing by releasing paracrine factors in this low-oxygen setting (Chen et al., 2014b; Lee et al., 2016). Vascularization is another critical factor in the healing and regenerative processes. MSCs release vasculogenic cytokines at higher rates, which boosts angiogenesis (Boomsma and Geenen, 2012). When EC and MSC conditioned media were tested under hypoxic conditions, the EC group showed significantly lower levels of vascular formation and VEGF expression than the MSC group (Zhang et al., 2012).

## 4 Conclusion

In conclusion, the potential of mesenchymal stromal cells (MSCs) in advancing wound healing therapies is truly remarkable. Their paracrine interactions play a pivotal role in expediting the healing process, promoting angiogenesis, and facilitating skin regeneration, making them a promising solution for cutaneous wounds. As researchers strive to enhance the therapeutic efficacy of MSC-based treatments, inducing hypoxia has emerged as a powerful tool to further unleash their capabilities.

By subjecting MSCs to hypoxic pre-conditioning, we can better equip them to withstand the challenges of the wound microenvironment, minimizing cell death and maximizing their beneficial effects on tissue repair. The future of wound healing appears brighter than ever as we delve deeper into the transformative potential of MSCs under hypoxic conditions. Embracing this approach not only holds promise for improving patient outcomes but also paves the way for more effective and efficient wound healing strategies.

By harnessing the power of MSCs in the context of hypoxia, we are forging a path towards advanced regenerative therapies that will enhance the lives of patients and promote overall skin health. The journey to harnessing the full regenerative potential of MSCs in wound healing has just begun, and its impact on medical practice is bound to be profound.
